# Multiomics characteristics of neurogenesis-related gene are dysregulated in tumor immune microenvironment

**DOI:** 10.1038/s41525-021-00202-y

**Published:** 2021-05-31

**Authors:** Ben Wang, Hai Mou, Mengmeng Liu, Zhujie Ran, Xin Li, Jie Li, Yunsheng Ou

**Affiliations:** 1 Department of Orthopedics, The First Affiliated Hospital of Chongqing Medical University, Chongqing, China; 2 Anhui No. 2 Provincial People’s Hospital, Hefei, China; 3 School of Public Health and Community Medicine, Wenzhou Medical University, Wenzhou, China; 4 Department of Respiratory and Critical Care Medicine, The First Affiliated Hospital of Chongqing Medical University, Chongqing, China; 5 Department of Oncology, The First Affiliated Hospital of Chongqing Medical University, Chongqing, China

**Keywords:** Cancer microenvironment, Data mining

## Abstract

The success of immunotherapy was overshadowed by its low response rate, and the hot or cold tumor microenvironment was reported to be responsible for it. However, due to the lack of an appropriate method, it is still a huge challenge for researchers to understand the molecular differences between hot and cold tumor microenvironments. Further research is needed to gain deeper insight into the molecular characteristics of the hot/cold tumor microenvironment. A large-scale clinical cohort and single-cell RNA-seq technology were used to identify the molecular characteristics of inflamed or noninflamed tumors. With single-cell RNA sequencing technology, we provided a novel method to dissect the tumor microenvironment into a hot/cold tumor microenvironment to help us understand the molecular differences between hot and cold tumor microenvironments. Compared with cold tumors, hot tumors highly expressed B cell-related genes, such as MS4A1 and CXCR5, neurogenesis-related miRNA such as MIR650, and immune molecule-related lncRNA such as MIR155HG and LINC00426. In cold tumors, the expression of genes related to multiple biological processes, such as the neural system, was significantly upregulated, and methylome analysis indicated that the promoter methylation level of genes related to neurogenesis was significantly reduced. Finally, we investigated the pan-cancer prognostic value of the cold/hot microenvironment and performed pharmacogenomic analysis to predict potential drugs that may have the potential to convert the cold microenvironment into a hot microenvironment. Our study reveals the multiomics characteristics of cold/hot microenvironments. These molecular characteristics may contribute to the understanding of immune exclusion and the development of microenvironment-targeted therapy.

## Introduction

The recent clinical successes of immunotherapy, including immune checkpoint inhibitors and adoptive cell therapy, represent a turning point in cancer treatment^1,2^. Clinical trials of anti-PD-1 for patients with melanoma have demonstrated substantial therapeutic responses. Despite these encouraging clinical results, only a fraction of patients can benefit from immunotherapy^3^.

Recent studies suggest that the phenotype of the tumor microenvironment (TME) is a critical factor influencing the efficacy of immunotherapy. From here, the tumor microenvironment can be broadly categorized as an inflamed (hot) or noninflamed (cold) tumor microenvironment^4,5^. The inflammatory tumor microenvironment was characterized by rich infiltration of immune cells. These tumors are correlated with significant tumor regression when treated by immunotherapy^6–10^. However, due to the lack of appropriate experimental methods, how different tumor cells shape their TME, thereby determining their response to therapy, remains a critical unsolved problem.

Here, based on single-cell RNA sequencing technology, a powerful tool to dissect the complexity of the tumor microenvironment^11–14^, we identified multiomics molecular alterations in the inflamed/noninflamed tumor microenvironment. Unexpectedly, we found that the multiomics characteristics of neurogenesis-related genes were dysregulated in a cold microenvironment. However, it is interesting that a similar discovery has been reported in recent studies. Balanis et al. reported that these neuroendocrine-related molecular characteristics were unexpectedly found in hematological malignancies and correlated with treatment resistance^15^. Pathologically, neuroendocrine tumors are a rare tumor subtype that originate from a variety of tissues, including small cell lung cancer and neuroendocrine prostate cancer^15^. Previous studies have revealed that neuroendocrine tumors exhibit an “immune desert” microenvironment^16^. Our results suggested that neuroendocrine-related molecular alterations may be more widespread than we previously thought, although most tumors cannot be classified into this subtype based on the recent pathological definition.

## Results

### Classifying the tumor microenvironment into inflamed or noninflamed with single-cell RNA-seq

**Fig. 1:**
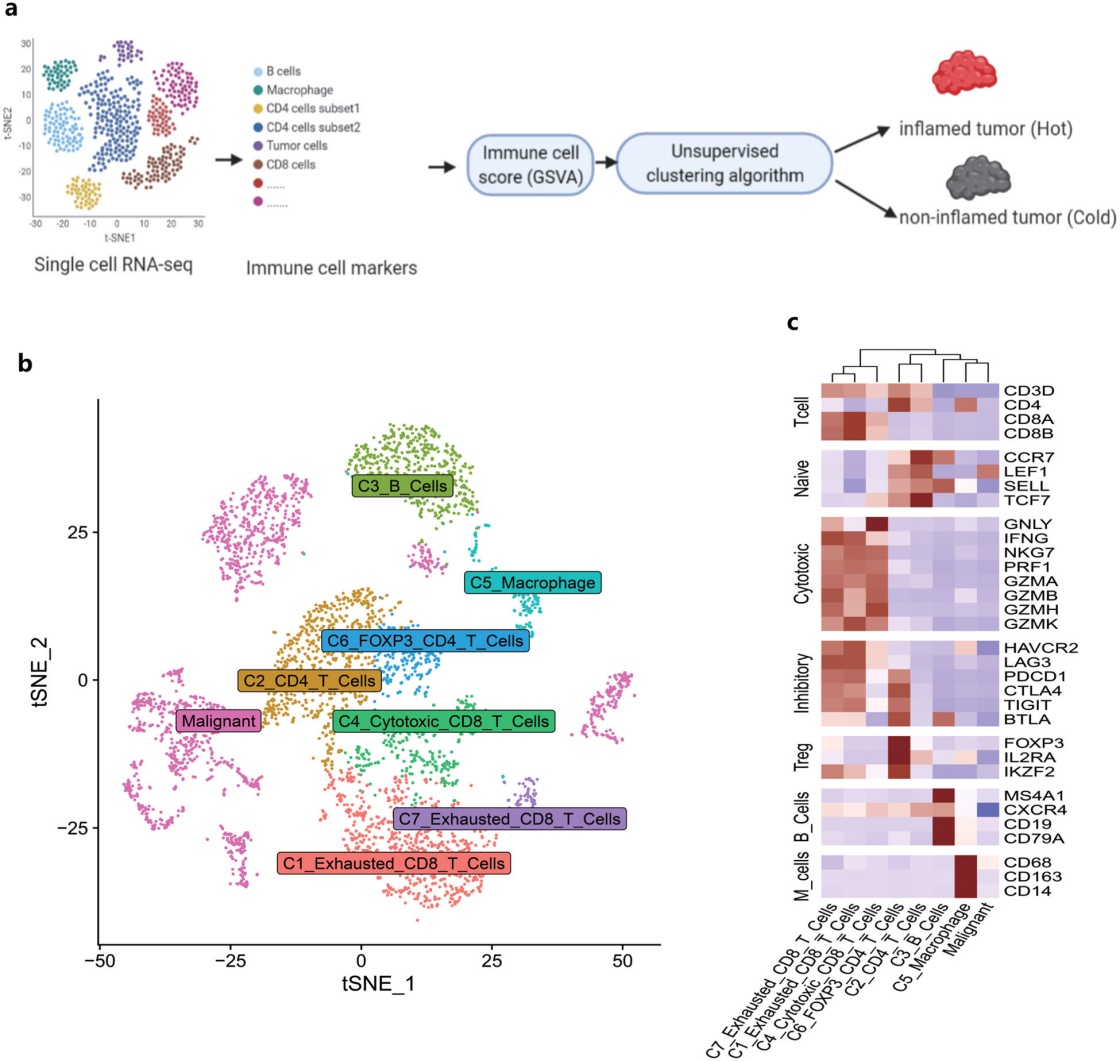
Acquisition of immune cell signatures. **a** Overview of the workflow design. **b** tSNE dimension plot of cells in tumors. Each dot represents a cell colored and labeled by inferred cell types. **c** Heatmap for cell-type annotation in single-cell RNA sequencing. The color represents the mean expression (log2 (TPM + 1)) of molecular markers (red: high, blue: low).

Before classifying the tumor microenvironment as hot/cold, we needed to obtain a set of tumor-infiltrating immune cell gene markers to estimate the immune cell infiltration of each tumor sample (Fig. 1a).

**Fig. 2:**
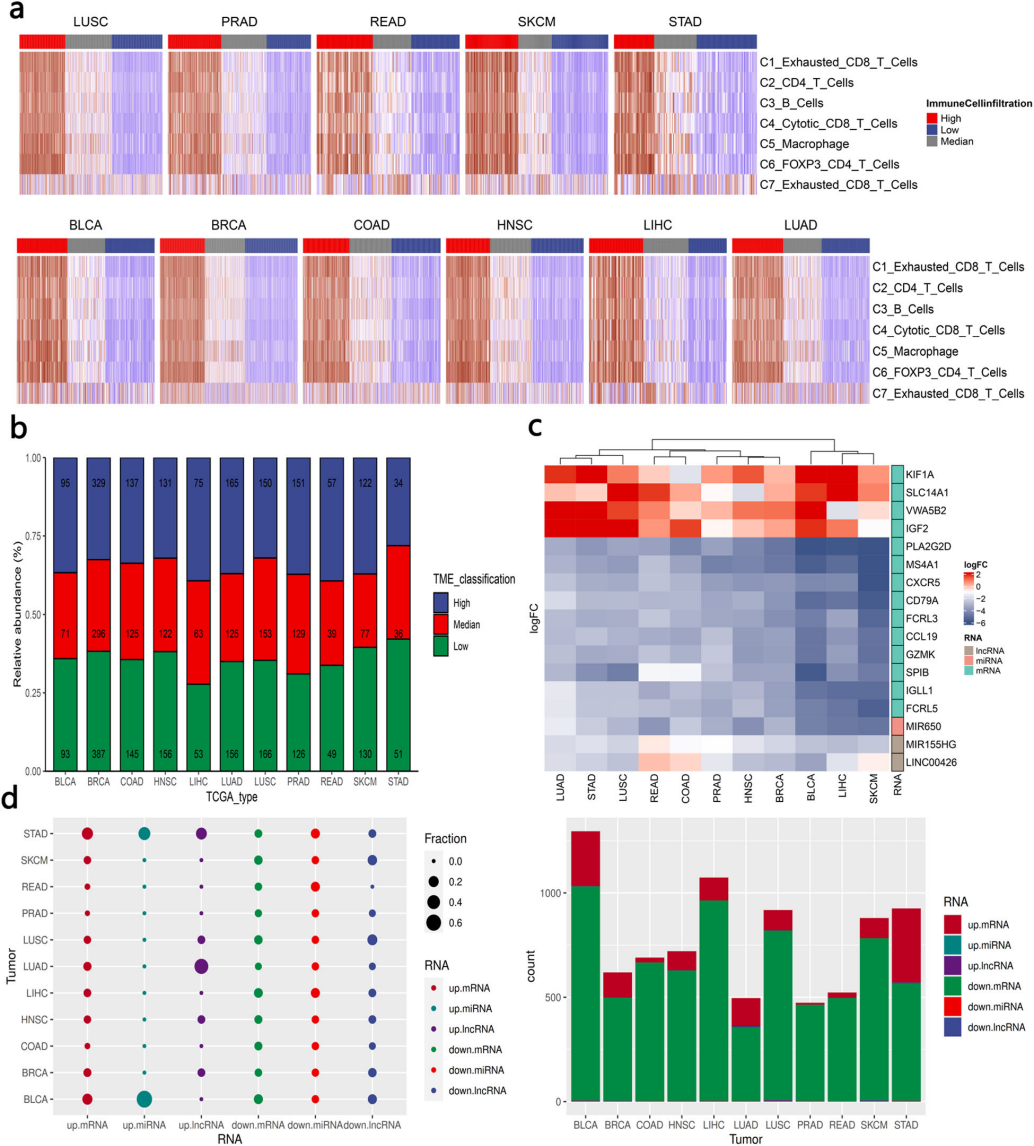
Unsupervised clustering identification of the cold/hot tumor immune microenvironment. **a** Heatmap for global expression (estimated with the GSVA algorithm) of immune cell markers in each TCGA tumor type. **b** Stacked bar plot for the proportion of immune microenvironment type in each tumor type. The number in each bar is the number of tumor samples assigned to the corresponding TME classification (high: inflamed, low: noninflamed, median: excluded). **c** Heatmap for the differentially expressed molecular characteristics (including mRNA, lncRNA, miRNA). The heatmap cell is colored according to the fold change of genes in differential expression analysis, where red represents genes that are upregulated in noninflamed tumors. **d** Overview of the molecular signature differences between inflamed and noninflamed TMEs. The fraction in the left panel represents the proportion of molecular characteristics from a tumor type among the molecular characteristics from all tumor types.

To generate robust immune cell gene markers to separate the tumor microenvironment into a hot/cold TME, we first performed single-cell RNA-seq analysis to identify immune cell-specific molecular markers (Fig. 1b, c and Supplementary Data 2). Then, these molecular markers were used as input for the GSVA algorithm^17^ to estimate the immune cell-level score of tumor samples. Finally, based on the unsupervised clustering pattern (immune cell scores) of tumor samples, we classified tumor samples into the high immune score (inflamed)/median immune score/low immune score (noninflamed). The unsupervised clustering results are shown in Fig. 2a. Tumor samples with median immune scores were excluded to avoid potential confounding factors (Fig. 2b). We also used methylomics data^18^ and copy number variant (CNV) data^19^ to validate the unsupervised clustering results (Supplementary Fig. 8).

Then, we compared RNA expression between the inflamed TME and the noninflamed TME, and significant alterations in RNA expression across different cancer types were observed. The number of differentially expressed RNAs varied across tumor types (upregulated (noninflamed-specific) mRNA ranging from 9 to 355; downregulated (inflamed-specific) mRNA ranging from 355 to 1024). Among them, downregulated mRNA (inflamed tumor-specific) represents the most striking signature that accounts for major differences between inflamed TME and noninflamed TME (Fig. 2d).

For example, several genes involved in B cell-associated immune processes were biased in most tumor types (Fig. 2c), including membrane-spanning 4-domain A1 (MS4A1), immunoglobulin lambda-like polypeptide 1 (IGLL1), B-cell antigen receptor complex-associated protein alpha chain (CD79A), C–X–C motif chemokine receptor 5 (CXCR5), and immune receptor translocation-associated protein 2 (FCRL5), suggesting that inflamed tumor types were also B cell-rich tumors, which has been proven to be a key factor determining sensitivity to immunotherapy^20,21^.

**Fig. 3:**
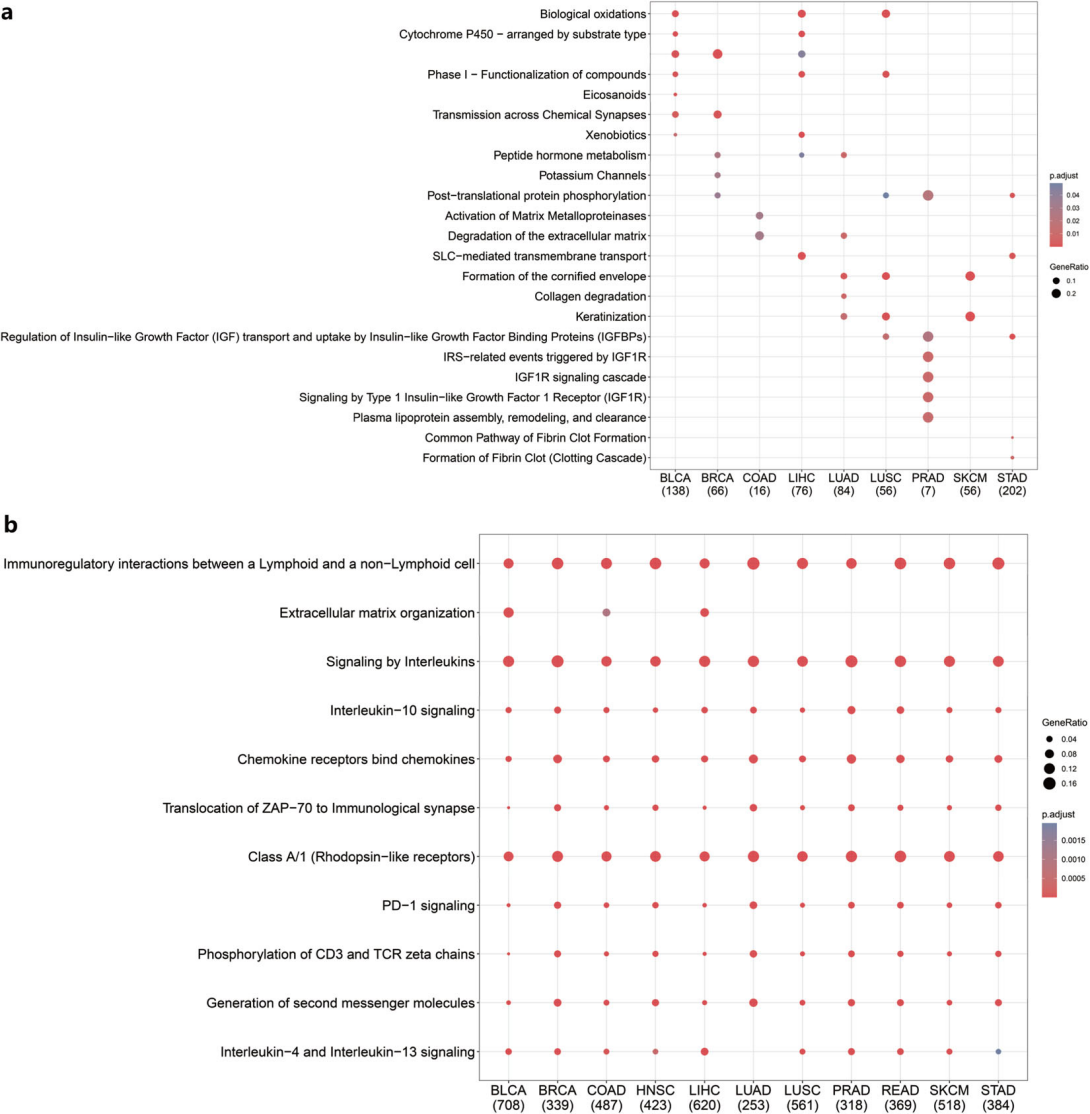
Gene function analysis of inflamed/noninflamed TME-specific mRNA (with Reactome datasets). **a** Enriched biological process for noninflamed TME-specific genes expressed across tumor types. **b** Enriched biological process for inflamed TME-specific genes expressed across tumor types. BLCA bladder urothelial carcinoma, BRCA breast invasive carcinoma, COAD colon adenocarcinoma, LIHC liver hepatocellular carcinoma, LUAD lung adenocarcinoma, LUSC lung squamous cell carcinoma, PRAD prostate adenocarcinoma, READ rectum adenocarcinoma, SKCM skin cutaneous melanoma, STAD stomach adenocarcinoma, HNSC head and neck squamous cancer (the number below the TCGA tumor type represents the number of identified genes in each category, the gene ratio represents the ratio of differentially expressed genes in the total pathway gene sets).

More systematic functional insight into the inflamed/noninflamed TME demonstrated that genes involved in the neuronal system, extracellular matrix degradation, biological oxidation, and IGFBP-associated pathways were more likely to be upregulated in the noninflamed TME but varied across tumor types (Fig. 3a). In contrast, enriched biological processes of inflamed TME-specific mRNA tended to be shared across tumor types, for example, signaling by interleukins (interleukin-4/13/10) and chemokines and PD-1 signaling, suggesting a recruiting and inhibitory tumor microenvironment for such inflamed tumors (Fig. 3b).

### Noncoding RNA pattern of inflamed and noninflamed TMEs

To further investigate the effect of the inflamed TME on noncoding RNA expression, we identified miRNAs and lncRNAs that were differentially expressed between the inflamed TME and the noninflamed TME. As shown in Fig. 2c, hsa-miR-650 was upregulated in the inflamed TME in most tumor types.

**Fig. 4:**
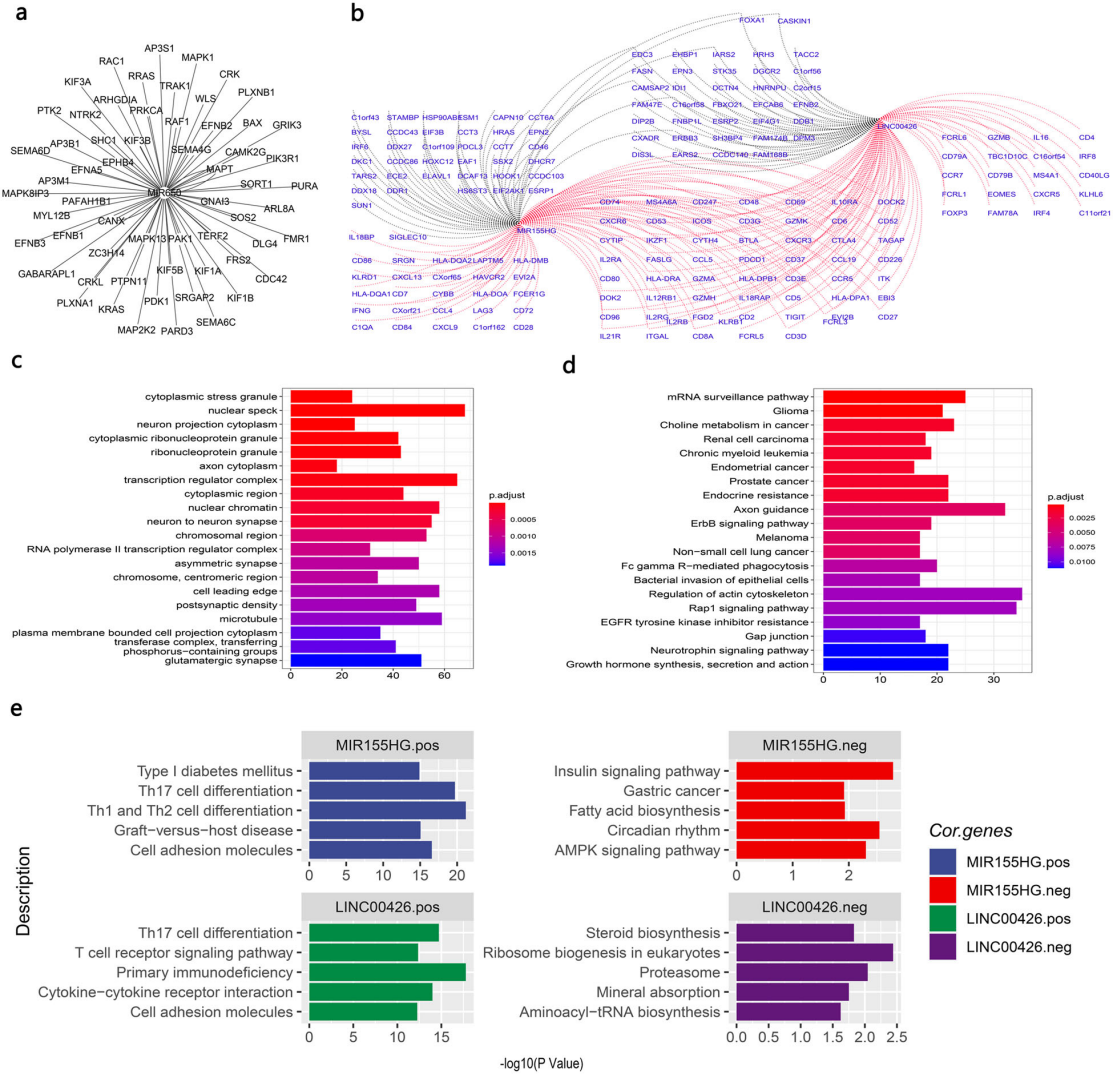
Noncoding RNA patterns of inflamed and noninflamed TMEs. **a** Potential targets of miR-650 (only genes annotated by neuronal pathways in **c** and **d** were visualized, and all potential targets are shown in Supplementary Data 1). **b** Coexpression network of identified lncRNAs. The red line represents a positive correlation, and the black line represents a negative correlation. **c** Gene ontology (GO) functional analysis of potential miRNA targets. **d** Functional analysis of miRNA targets with the Kyoto Encyclopedia of Genes and Genomes (KEGG) database. **e** Functional analysis of lncRNA coexpressed genes was performed with the Kyoto Encyclopedia of Genes and Genomes (KEGG) database. The left panel shows the functions of lncRNA positively correlated genes. The right panel shows the functions of lncRNA negatively correlated genes.

Further analysis revealed that hsa-miR-650 may inhibit the neurotrophic signaling pathway and axon guidance (Fig. 4c, d) by inhibiting nerve-associated receptors (such as neurotrophic receptor tyrosine kinase 2 (NTRK2) and GABA type A receptor-associated protein-like 1 (GABARAPL1)) and downstream molecules (such as CRK, CRKL, RRAS, PTK2, KRAS, and ephrin (EPH) family members: EPHB1, EPHB4, EFNB1/2/3, and EFNA5) (Fig. 4a).

In terms of lncRNAs, lncRNAs including MIR155HG and LINC00426 were upregulated in the inflamed TME (Fig. 2c).

Coexpression analysis revealed that these lncRNAs were positively associated with T-helper cell differentiation and cytokine signaling pathways (Fig. 4e), such as multiple immune markers, including cytotoxic markers: KLRD1, GZMB, GZMA, and GZMH; immune coinhibitory and costimulatory molecules: PDCD1, HAVCR2, CTLA4, TIGIT, FOXP3, and ICOS; and chemokine receptors and ligands: CXCR3/6, CXCL9/13, CCL4/5/7/19, and CCR7 (Fig. 4b). These coexpression analysis results revealed that these lncRNAs may have the potential to shape the tumor immune microenvironment by interacting with immune T-helper cell differentiation and cytokine signaling pathways.

### Methylation pattern of inflamed and noninflamed TMEs

**Fig. 5:**
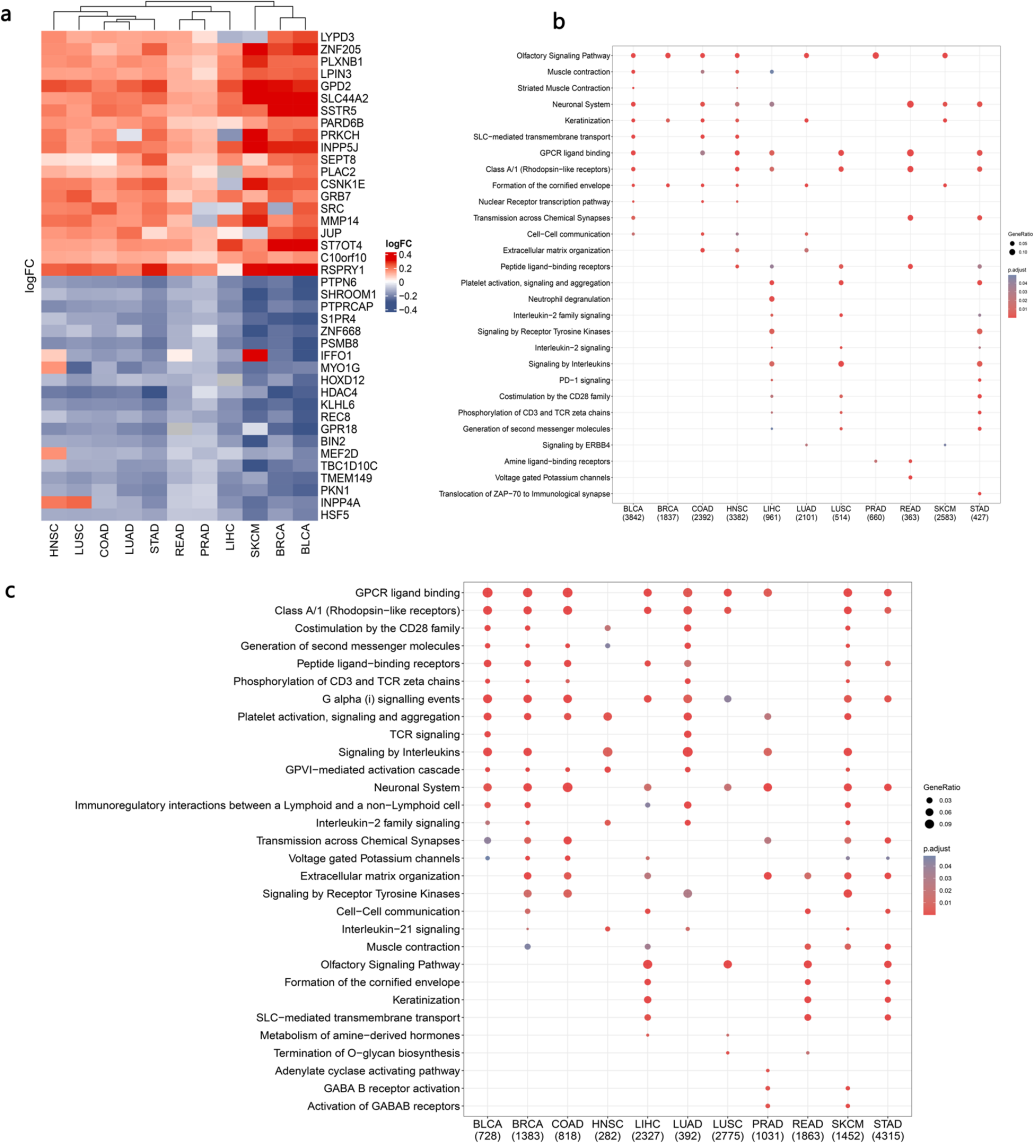
Reactome pathway analysis of inflamed/noninflamed-specific differently methylated regions. **a** Heatmap for differently methylated probes. Red represents inflamed tumor-specific hypermethylated probes and noninflamed tumor-specific hypomethylated probes. Gray represents noninflamed tumor-specific hypermethylated probes and inflamed tumor-specific hypomethylated probes. **b** Enriched reactome pathways for noninflamed tumor-specific hypomethylated genes across TCGA tumor types. **c** Enriched reactome pathways for inflamed tumor-specific hypomethylated genes across TCGA tumor types. The number (below **b** and **c**) refers to the number of dysregulated genes (up or down) in each tumor type.

Alteration of DNA methylation is an important epigenetic mechanism that results in the dysregulation of RNA. Therefore, we further investigated the alteration of DNA methylation between inflamed and noninflamed tumors. As shown in Fig. 5a, b, the promoters of several genes involved in oncogenesis biological processes were hypomethylated in noninflamed tumors, which suggests that the abnormal hypomethylation of these genes may confer invasive and metastatic abilities to noninflamed tumors; for example, cell-matrix adhesion-related genes: proto-oncogene c-Src (SRC), GPI-anchored metastasis-associated protein homolog (LYPD3); and invasion- and metastasis-associated molecules: matrix metallopeptidase 14 (MMP14).

Interestingly, consistent with the mRNA and noncoding RNA results, we found that different methylated regions (hypomethylated in noninflamed tumors) were related to the neuronal system and GABAB receptor-associated pathways, which suggested that methylation-level dysregulation of neuronal genes may be relevant to the formation of an inflamed/noninflamed tumor microenvironment (Fig. 5c). In terms of inflamed tumors, multiple immune-associated molecular pathways were hypomethylated, including costimulation by the CD28 family, TCR signaling, and interleukin-21/2 signaling (Fig. 5d).

### Screening for potential drugs that may convert noninflamed tumors to inflamed tumors

Despite the limited understanding of why the response rate of immunotherapy is unsatisfactory, it is increasingly clear that combination immunotherapy with classical therapy is the most feasible way to improve the response rate of immunotherapy^4^.

Combination immunotherapy with classical therapy is considered the most feasible way to improve the response rate of immunotherapy^4^. However, limited by classical methods, a systemic understanding of hot/cold tumor microenvironment genomic characteristics is still limited, which constrains related drug discovery with big data^22^.

**Fig. 6:**
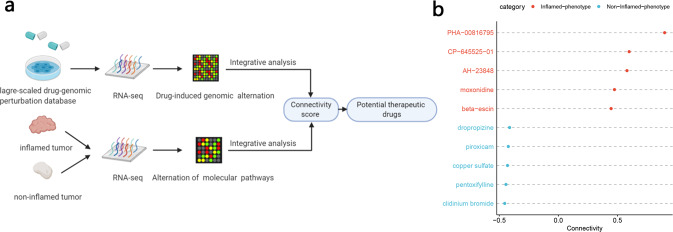
Analysis combining pharmacogenomic perturbation database screens of multiple drugs that have the potential to promote inflamed or noninflamed immunophenotypic switching. **a** The schematic plot shows the workflow for identifying potential drugs that can shape the tumor microenvironment. **b** The dot plots show potential adjuvant drugs that may contribute to favorable immunophenotype transformation. Red: drugs that may induce systematic transcriptomic alternation from a noninflamed TME to an inflamed TME. Blue: opposite drugs. Connectivity is a score calculated by the PharmacoGx package based on the CMAP algorithm, and it represents the drug’s potential to convert the transcriptomic characteristics of “cold” tumors into “hot” tumors.

Here, based on these molecular characteristics and an efficient and widely used computational drug-genomic method^23–25^, we identified multiple drugs that may have the potential to convert a noninflamed environment into an inflamed tumor environment (Fig. 6a). These results may be helpful for developing novel combination immunotherapeutic strategies, and further clinical trials or experiments are also needed to validate these results.

As shown in Fig. 6b, the histone deacetylase inhibitor PHA00816795^26^, the active compound β-escin from Aesculus hippocastanum L. seeds, the peripheral sympathetic nerve activity inhibitor moxonidine^27,28^, and the PGE2 receptor antagonist AH23848^29^ were predicted to be promising drugs. Although these drugs have totally different molecular targets, as these results predicted, the potential of these drugs in immune modulation has been supported by an increasing number of recently published articles. For example, histone deacetylase inhibitors (HDACs) are regarded as promising drug candidates by combining them with immunotherapy^30–33^, and moxonidine^34–37^, β-escin^38,39^ and AH23848^40–46^ have shown potential in cytokine signaling modulation and inflammation regulation in published reports. However, whether these drugs can directly affect antitumor immunity in vivo and whether they can enhance the effect of immunotherapy still need to be further explored.

In total, our method provides an efficient way to identify robust molecular signatures of the tumor microenvironment. These molecular characteristics may serve as efficient resources and provide an opportunity for related drug discovery.

### The identified noncoding RNAs and immunophenotypes correlate with tumor patient prognosis

**Fig. 7:**
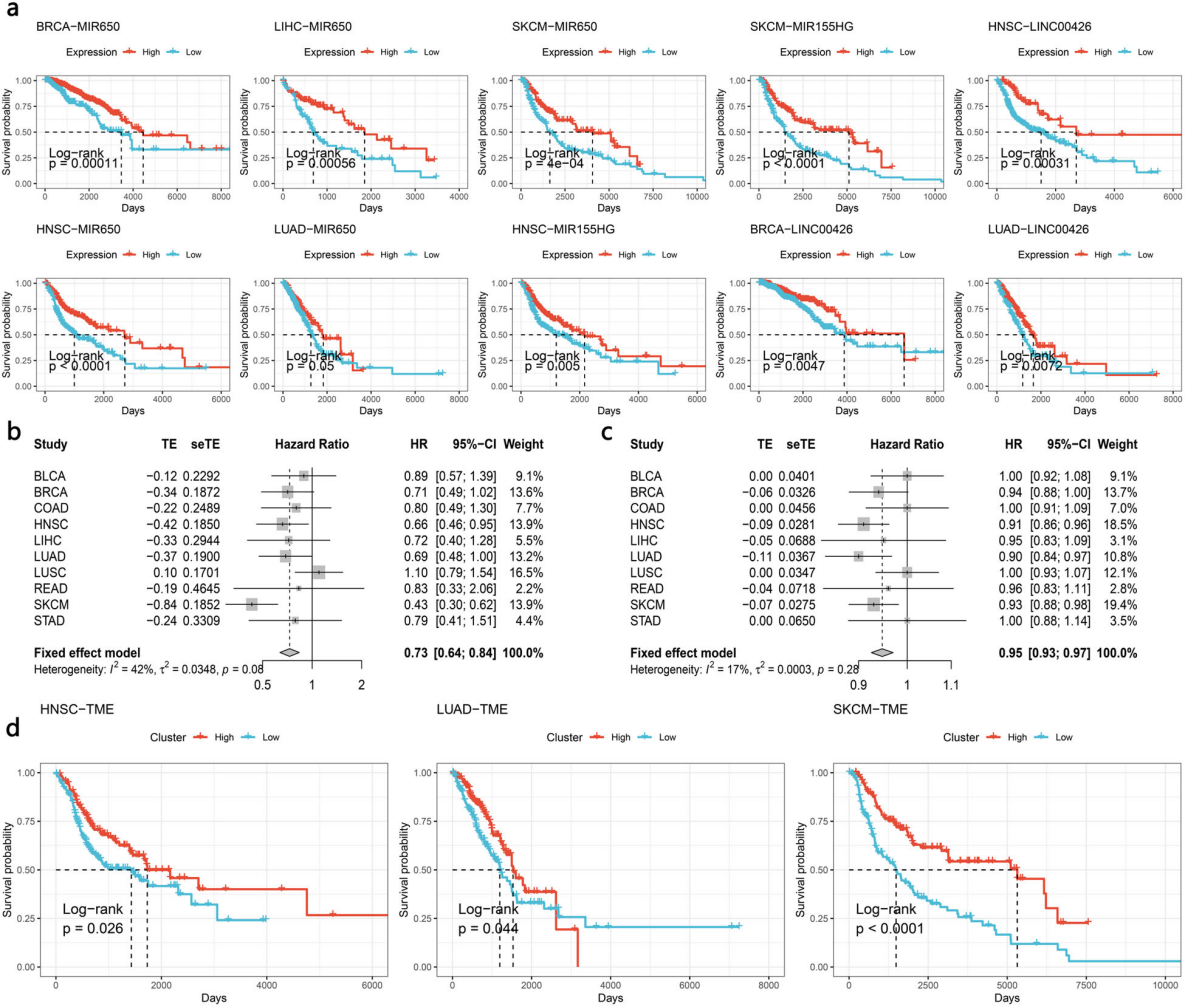
Prognostic role of immunophenotypes and noncoding RNA. **a** Kaplan–Meier plots show the overall survival rate for noncoding RNAs. The *P* value was calculated using the log-rank model. **b**, **c** Forest plots show hazard ratios for identified immunophenotypes. The hazard ratio of **b** was calculated by a single variable Cox model. **c** is from the multiple variable cox model. **d** Kaplan–Meier plots show the overall survival rate for the identified immunophenotypes. The *P* value was calculated using the log-rank model.

To explore the prognostic significance of the identified noncoding RNAs and immune subtypes, univariate/multivariate Cox models and Kaplan–Meier analyses were performed. Kaplan–Meier analysis revealed that these noncoding RNAs (LNC00426, MIR155HG, and MIR650) were positively associated with better overall survival (OS) in BRCA, LUAD, HNSC, and SKCM (Fig. 7a). The univariate/multivariate Cox model also validated that LNC00426, MIR650, and miR155HG were independently and significantly correlated with the prognosis of tumor patients. These noncoding RNAs may serve as pan-cancer prognostic markers (Supplementary Tables 1–4, LNC00426: positively correlated with the prognosis of BLCA, BRCA, HNSC, and LUAD patients, average hazard ratio (HR) 0.49–0.74; miR155HG: positively correlated with BLCA, HNSC, and SKCM, HR: 0.74–0.77; miR650: positively correlated with BRCA, COAD, HNSC, LIHC, LUAD, LUSC, READ, and SKCM, HR: 0.91–0.93).

Considering the favorable role of these noncoding RNAs and their potential to regulate the tumor immune microenvironment, further investigation of these noncoding RNAs may be helpful to elucidate the role of noncoding RNAs in the tumor immune microenvironment.

We further investigated the prognostic role of immunophenotypes across tumor types. As shown in Fig. 7b, the pooled hazard ratio of the inflamed TME was 0.73 (univariate Cox model 95% CI 0.64–0.84) or 0.95 (multivariate Cox model, 95% CI 0.93–0.97). Kaplan–Meier analysis also suggested that an inflamed phenotype was associated with better OS (Fig. 7c).

## Discussion

Neuroendocrine-related molecular characteristics have been reported in some tumors, but little is known about how the tumor genome shapes it^16,47^. Our results may provide some mechanistic insights for this persistent unsolved problem. Here, we found that neurogenesis-associated multiomics characteristics were significantly dysregulated between noninflamed and inflamed tumor microenvironments, which is interesting and complements previous reports. For example, Nikolas G Balanis et al. reported that a neuroendocrine phenotype existed in multiple hematological malignancies, and transdifferentiation into a neuroendocrine phenotype conferred treatment resistance to tumors^15^. Our results indicated that neurogenesis-related genes were also dysregulated in a variety of solid tumors and may be modified in various ways, including noncoding RNA and methylation. For example, miR-650, which is downregulated in noninflamed tumors, may enhance the expression of neurogenesis-related genes in the cold tumor microenvironment. The promoters of multiple neurogenesis-associated genes were hypomethylated in multiple noninflamed tumors^48^.

Our studies also revealed that lncRNAs may act as general regulators in the inflamed tumor microenvironment. The expression of MIR155HG and LINC00426 was altered in pan-cancer, and the coexpressed genes of MIR155HG and LINC00426 were involved in multiple immune-associated biological processes, including immune cell differentiation and exhausted/activated T-cell genetic programs, indicating that these lncRNAs might assist in the maintenance of the inflamed tumor microenvironment across tumor types, especially MIR155HG, which is an inflamed TME-specific gene in all analyzed tumor types.

This study has some limitations. First, validation in other tumor types or a large cohort is warranted in further studies. Second, TCGA does not provide direct information on the tumor microenvironment. Therefore, we had to indirectly infer the relative score of the immune composition as described in previous studies^49^. Third, our study provided a comprehensive catalog of molecular alterations, but we could not further investigate the role of the identified molecule due to the lack of funding support and the experimental environment. Therefore, further studies are necessary to elucidate the detailed role of these molecular characteristics.

In conclusion, our study identified multiple molecular differences between inflamed and noninflamed TMEs. These results provide comprehensive insights into the inflamed tumor microenvironment-related molecular mechanisms and have profound clinical implications. These results may help to optimize current combination immunotherapy to benefit more tumor patients.

Nevertheless, our study calls attention to the need to include tumor microenvironment status in future clinical trials.

## Methods

### Clinical cohort and multiomics data for TCGA samples

RNA sequencing datasets, including mRNA expression, miRNA, and lncRNA, were downloaded from the GEO database with accession number GSE62944^50^. Healthy controls of GSE62944 were excluded in this study. Updated clinical data and DNA methylation data were downloaded from *TCGAbiolinks*^51–53^. The raw count data of RNA sequencing were normalized and quantitated by the edgeR package^54^. The immunotherapy clinical cohort was from Riaz et al.^55^ and Cloughesy et al.^56^. The clinical data of patients involved in this study was based on open-Access database, so the ethical declaration is not appliable. But patients in these databases have obtained ethical approval, all information about ethical approval of these open-Access databases can be obtained from GDC portal (https://docs.gdc.cancer.gov/).

### Identifying genetic signatures of immune cells from single-cell RNA sequencing data

Raw single-cell RNA sequencing data were downloaded from the GEO database with accession number GSE72056^57^. This dataset contains 4645 single cells isolated from 19 patients and profiles of immune and malignant cells within the tumor microenvironment. We applied the Seurat^58^ package to normalize the data and identify differentially expressed genes. Cells with unique feature counts over 10,000 or less than 1000 (these thresholds were determined by the QC metrics of Seurat) and features detected in less than three cells were excluded. tSNE was used for dimension reduction. Each cell is represented as a dot in a two-dimensional tSNE plane. The annotation of cells was annotated based on Guo et al.^59^ and the CellMarker database^60^. Gene signatures of immune cells were selected according to the following workflow (taking B cells as an example). First, markers of B cells should be positively correlated with B cells and statistically significant (detailed criteria: compared to malignant and other immune cells, log fold change >0 and adjusted *P* value <0.05). Then, to make sure that these markers are as highly expressed as possible in B cells and as lowly expressed as possible in malignant and other immune cells, we sorted the markers identified in the above step in decreasing order of the absolute value of PCT_B_cells_-PCT _malignant_ and the absolute value of PCT_B_cells_-PCT _malignant_ (PCT is an index calculated by the Seurat package^58^, which represents the expression percentage of a molecular marker in a cell subtype). The top 1% of gene markers in the sorted list with the highest absolute value of PCT difference were regarded as B cell-specific molecular markers.

### Classification of TME phenotypes across different tumor types

To investigate the molecular characteristics of inflamed or noninflamed immunophenotypes, immune cell signatures identified in the above single-cell RNA sequencing data were used as input for the GSVA algorithm^17^ to calculate the immune score for each immune cell type. The GSVA algorithm has been proven to be an efficient way to reveal the characteristics of the tumor microenvironment^49^. Then, tumor samples were classified into the low immune score (noninflamed), median immune score, and high immune score (inflamed) TME groups based on the unsupervised clustering pattern of the immune score by optCluster^61^. To avoid confounding factors from potential mixtures, we excluded samples from the median immune score from further analysis. We also used methylation data (with the MethylCIBERSORT algorithm)^18^ and CNV data (with the ABOSULTE algorithm)^19^ to estimate immune cell infiltration.

### Identification of molecular differences between inflamed and noninflamed tumors

Then, we compared the molecular data between these two groups to identify molecular differences.

The EdgeR^54^ package was used to perform differential gene expression analyses with the raw count matrix of TCGA data. The statistically significant criteria for each molecular characteristic were as follows: molecular characteristics (mRNA, miRNA, and lncRNA) with an absolute value of the log fold change >1.5 and FDR *P* value < 0.05 were considered significant. To ensure that these molecules could represent pan-cancer dysregulated characteristics, we only included molecular characteristics differentially expressed in at least four tumor types. We next calculated Spearman’s correlation coefficient between identified noncoding RNAs and coding RNAs. Potential coding RNA targets were selected based on the following criteria: lncRNAs: significantly correlated mRNAs (FDR < 0.05) of lncRNAs were ranked with the average absolute value of Spearman’s correlation coefficient in decreasing order, and the top 1% of mRNAs (with the highest correlation coefficient) in this list were regarded as potential targets of lncRNAs. miRNAs: potential targets of miRNAs^62^ (from fourteen miRNA-mRNA interaction databases) and negatively correlated with the expression of miRNA (Spearman’s correlation coefficient <0 and FDR < 0.05). Functional enrichment analysis (KEGG, Reactome, and GO) of potential targets was performed to contribute to the mechanistic understanding of identified noncoding RNAs^63^.

In methylation analysis, only CpG probes mapped at promoter regions (e.g., TSS1500, TSS200, 5’ UTR, 1stexon) were included in this analysis. Differently expressed regions were identified by a champ^64^ pipeline based on the following criteria^65,66^: (1) adjusted *P* value <0.05; and (2) absolute value of beta-value differences >0.1.

### Identification of potential drugs that convert a nonimmune tumor into an inflamed tumor

Combination immunotherapy is considered the most efficient way to aid current immunotherapy. Here, transcriptomic differences between inflamed and noninflamed tumors were used as an input to calculate the connectivity score between transcriptomic differences and drug-induced genomic alterations. Drug-induced systematic genomic alterations were obtained from the CMAP database^67^. The data download and connectivity score calculation were performed with PharmacoGx^68^.

### Statistical analysis

The chi-square test was used for statistical tests of categorical variables. Kaplan–Meier analysis and log-rank tests were used to test the survival differences between identified groups. The optimal cutoff point of continuous variables was determined by the surv_cutpoint function in the survminer package^69^.

The univariate Cox proportional hazard ratio was used to calculate the hazard ratio of the factor of interest. The multivariate Cox proportional hazard model was used to test the independence of variates. All statistical analyses were performed by R 3.61.

### Reporting summary

Further information on research design is available in the Nature Research Reporting Summary linked to this article.

## Supplementary information

Supplementary Information

Supplementary Data 1

Supplementary Data 2

Reporting Summary

## Data Availability

Raw and processed sequencing data have been deposited in the Gene Expression Omnibus (GEO) database under the accession number GSE62944, GSE72056. All publicly available datasets can be found on the corresponded website (TCGA: https://docs.gdc.cancer.gov/) and published open access articles (Riaz et al.^55^ and Cloughesy et al.^56^). All other relevant data supporting the key findings of this study are available within Supplementary Information files or from the corresponding author upon reasonable request.

## Code availability

Data analysis and graphical representation were performed using custom R scripts and publicly available packages as denoted in the text. All scripts are available upon request.
