# Supplementary material for: Multiomics characteristics of neurogenesis-related gene are dysregulated in tumor immune microenvironment
Source: NPJ Genom Med. 2021 May 31;6:37. doi: 10.1038/s41525-021-00202-y (PMC8166819; doi:10.1038/s41525-021-00202-y)
Supplement: Supplementary file 1 — Supplementary Information [file 41525_2021_202_MOESM1_ESM.pdf]

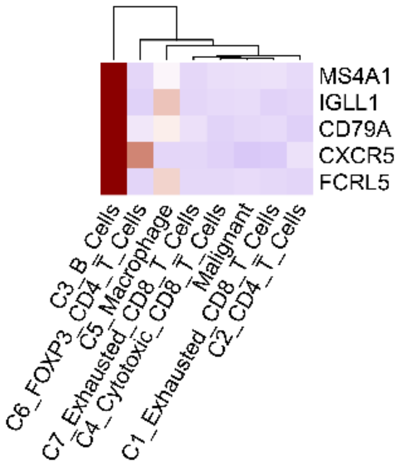

Supplementary Figure.1 Heatmap for the expression of interested genes in immune cells. the color referred to the mean expression (Log(TPM+1)) of gene.

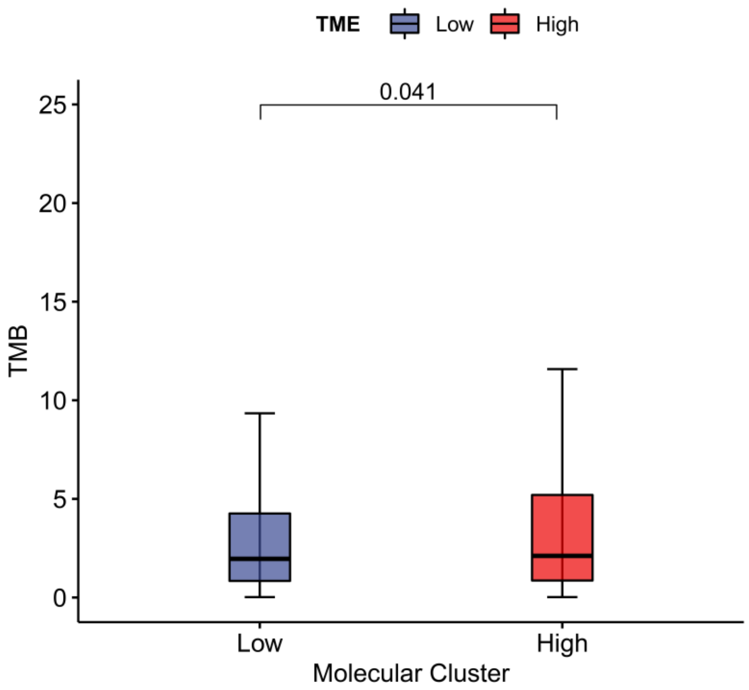

Supplementary Figure.2 The boxplot for tumor mutation burden (TMB) in each TME classification (inflamed: High vs non-inflamed: Low). The text on the line is the p value calculated by student t-test. Tumor mutation burden was estimated with maftools R package[1].

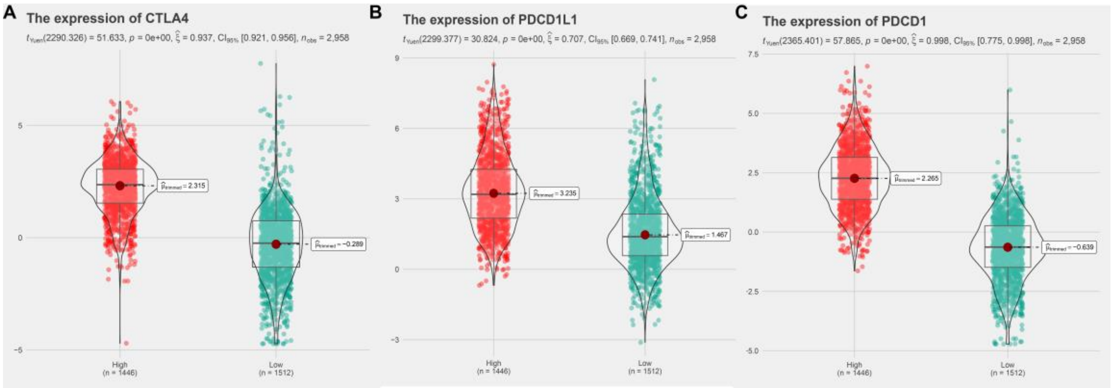

Supplementary Figure.3 The expression of immunotherapeutic responsive markers in each TME type. The y axis represents the expression (Log(CPM+1)) of these genes.

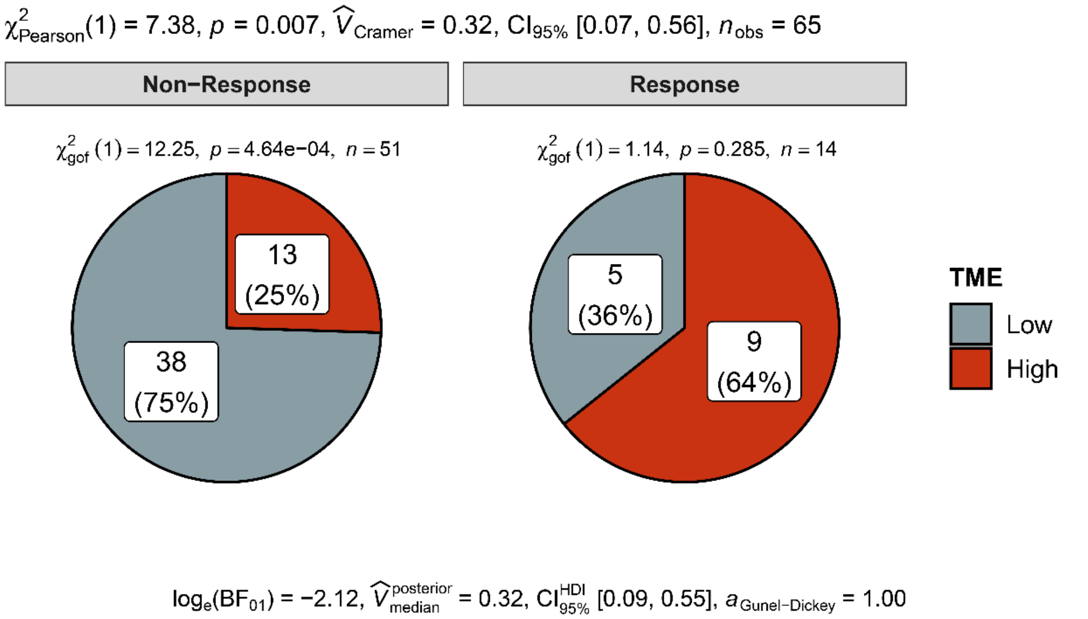

Supplementary Figure.4 The pie chart shows the proportion of High (inflamed) and Low (non-inflamed) tumor microenvironment in immunotherapeutic responders and non-responders. The immunotherapy clinical cohort was from Riaz et al[2].

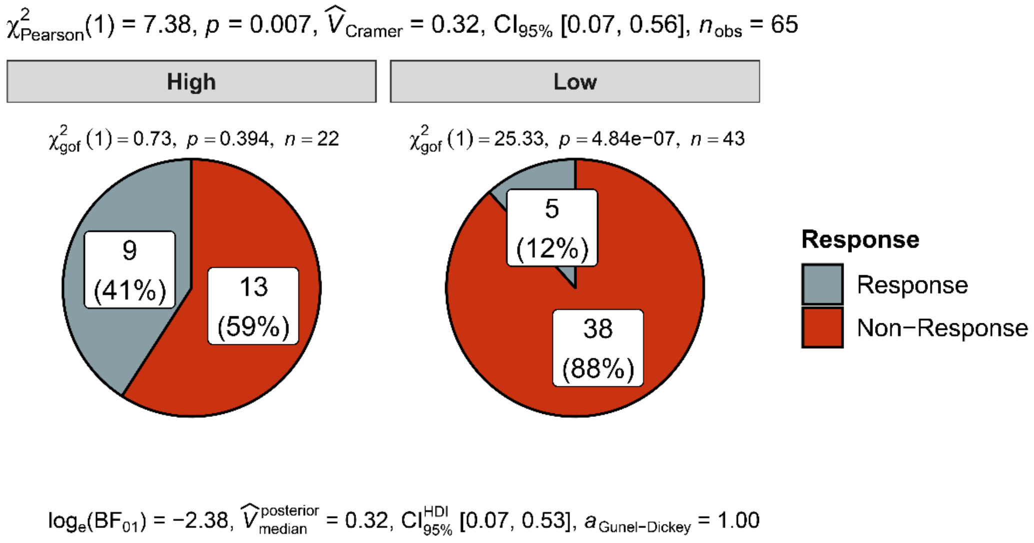

Supplementary Figure. 5 The pie chart for immunotherapeutic responsive rate in High (inflamed) and Low (non-inflamed) tumor microenvironment. The immunotherapy clinical cohort was from Riaz et al[2].

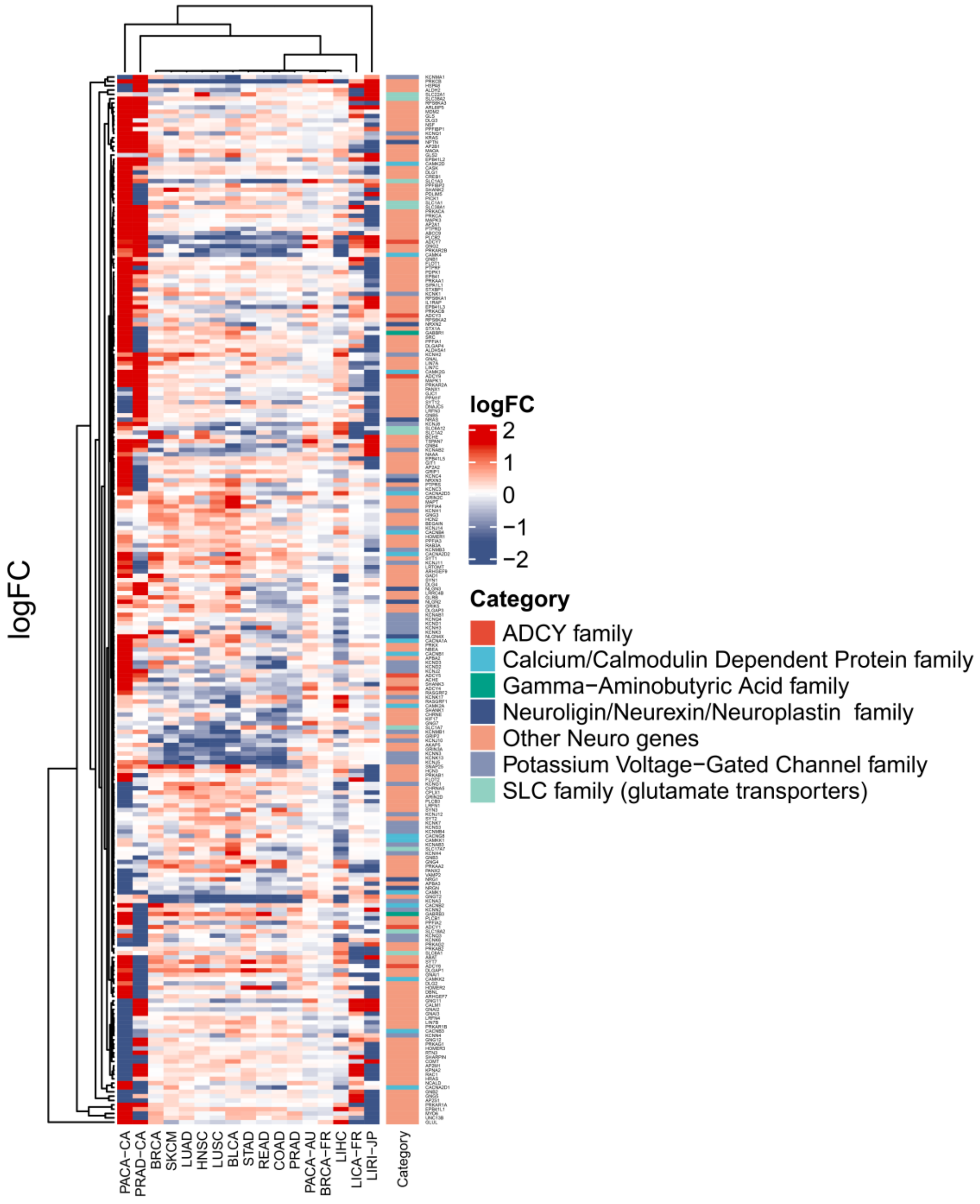

Supplementary Figure.6 Neuronal system associated genes were also dysregulated between inflamed and non-inflamed tumor microenvironment in International Cancer Genome Consortium[3] (ICGC) derived tumor samples.

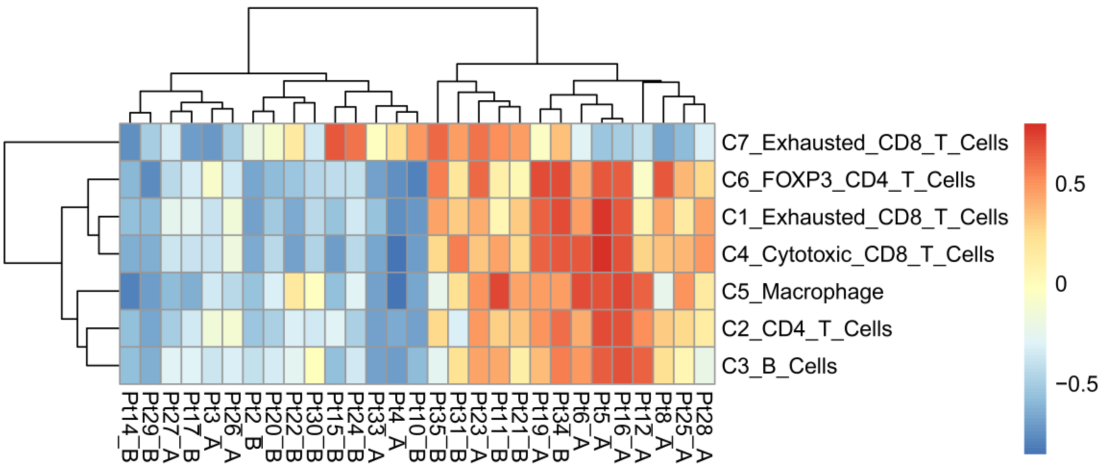

Supplementary Figure.7 Heatmap for immune cell scores (GSVA score for immune cell makers) for individual patients in Cloughesy's clinical cohort[4]. "A" denotes patient in neoadjuvant group; "B" denotes patient in adjuvant-only group. Blue represents low GSVA score; red represents high GSVA score.

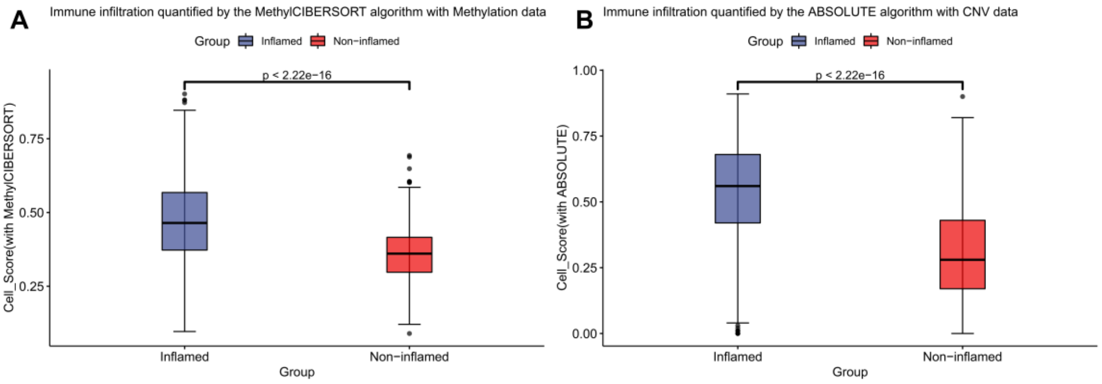

Supplementary Figure.8 MethylCIBERSORT algorithm and ABSOLUTE algorithm validated that the total immune cell infiltration of inflamed TME is higher than non-inflamed TME. A). The boxplot for total immune cell infiltration quantified by MethylCIBERSORT algorithm[5]. **B).** The boxplot for total immune cell infiltration quantified by ABSOLUTE algorithm[6]. The raw data from MethylCIBERSORT and ABSOLUTE algorithm was uploaded in Zenodo (https://zenodo.org/record/4643003#.YGEs8dLiuuU).

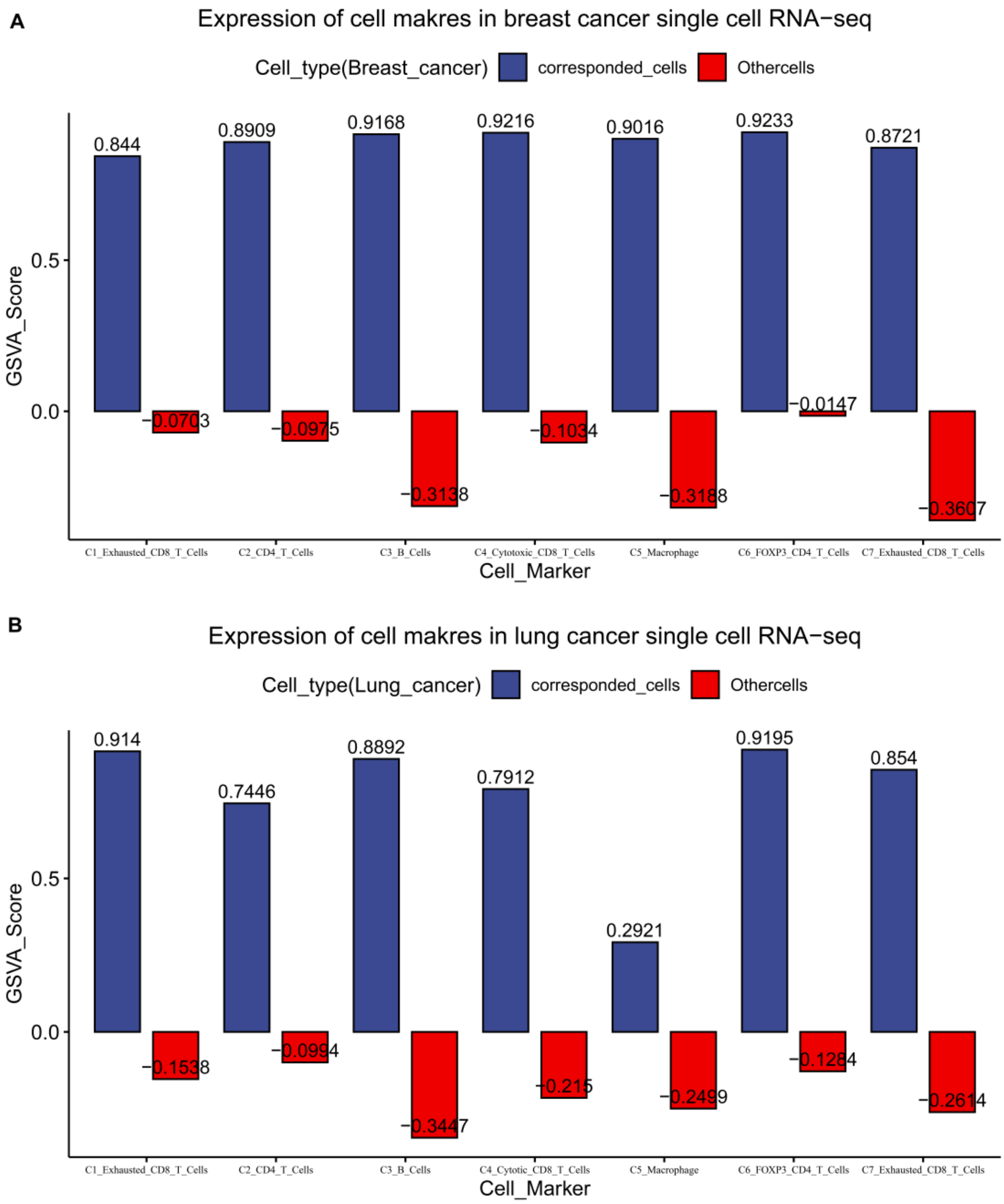

Supplementary Figure. 9 The GSVA score of immune cell markers in lung cancer/breast cancer derived immune cells. The blue represents same immune cell type in lung/breast cancer, the red represents immune cells and malignant cells (except for the same (corresponded) cell type). The lung cancer and breast cancer single cell RNA sequencing datasets were from Qian et al[7].

	**Single.HR (95% CI for HR)**	**Single.p.value**	**Multi.HR (95% CI for HR)**	**Multi.p.value**
BLCA	0.99 (0.93-1)	0.71	1 (0.94-1.1)	1

**BRCA**	0.92 (0.87-0.97)	0.0044	0.94 (0.88-0.99)	0.025
COAD	0.97 (0.9-1)	0.42	0.97 (0.9-1)	0.43
**HNSC**	0.92 (0.88-0.96)	0.00038	0.91 (0.87-0.96)	0.00016
**LIHC**	0.91 (0.82-1)	0.048	0.91 (0.82-1)	0.044
**LUAD**	0.93 (0.87-1)	0.043	0.93 (0.87-0.99)	0.035
LUSC	1 (0.97-1.1)	0.35	1 (0.97-1.1)	0.4
READ	0.92 (0.78-1.1)	0.31	0.94 (0.81-1.1)	0.44
**SKCM**	0.93 (0.89-0.98)	0.0029	0.95 (0.9-0.99)	0.025
STAD	1 (0.91-1.1)	0.78	1 (0.91-1.1)	0.81

Supplementary table 1. Single/Multi-variable cox test results for MIR650

Supplementary table 2. Single/Multi-variable cox test results for MIR155HG

	**Single.HR (95% CI for HR)**	**Single.p.value**	**Multi.HR (95% CI for HR)**	**Multi.p.value**
**BLCA**	0.74 (0.6-0.92)	0.0069	1 (0.94-1.1)	1
BRCA	0.92 (0.77-1.1)	0.33	0.94 (0.88-0.99)	0.025
COAD	1 (0.8-1.3)	0.79	0.97 (0.9-1)	0.43
**HNSC**	0.77 (0.64-0.93)	0.0068	0.91 (0.87-0.96)	0.00016
LIHC	1 (0.78-1.4)	0.82	0.91 (0.82-1)	0.044
LUAD	0.89 (0.76-1.1)	0.18	0.93 (0.87-0.99)	0.035
LUSC	0.97 (0.84-1.1)	0.71	1 (0.97-1.1)	0.4
READ	0.73 (0.37-1.4)	0.35	0.94 (0.81-1.1)	0.44
**SKCM**	0.75 (0.67-0.85)	5.20E-06	0.95 (0.9-0.99)	0.025
STAD	0.79 (0.59-1.1)	0.12	1 (0.91-1.1)	0.81

Supplementary table 3. Single/Multi-variable cox test results for LINC00426

	**Single.HR (95% CI for HR)**	**Single.p.value**	**Multi.HR (95% CI for HR)**	**Multi.p.value**
**BLC**	0.66 (0.44-0.98)	0.041	1 (0.94-1.1)	1

**A**				
**BRCA**	0.7 (0.51-0.95)	0.024	0.94 (0.88-0.99)	0.025
COAD	1 (0.71-1.5)	0.89	0.97 (0.9-1)	0.43
**HNSC**	0.49 (0.34-0.73)	0.00036	0.91 (0.87-0.96)	0.00016
LIHC	0.57 (0.26-1.3)	0.17	0.91 (0.82-1)	0.044
**LUAD**	0.74 (0.56-0.98)	0.034	0.93 (0.87-0.99)	0.035
LUSC	1 (0.82-1.3)	0.79	1 (0.97-1.1)	0.4
READ	0.95 (0.45-2)	0.9	0.94 (0.81-1.1)	0.44
SKCM	0.86 (0.74-1)	0.053	0.95 (0.9-0.99)	0.025
STAD	0.84 (0.58-1.2)	0.34	1 (0.91-1.1)	0.81

Supplementary table 4. Single/Multi-variable cox test results for TME clasification

	**Single.HR (95% CI for HR)**	**Single.p.value**	**Multi.HR (95% CI for HR)**	**Multi.p.value**
BLCA	0.89 (0.57-1.4)	0.6	1 (0.94-1.1)	0.96
BRCA	0.71 (0.48-1)	0.085	0.94 (0.88-1)	0.066
COAD	0.8 (0.49-1.3)	0.37	1 (0.92-1.1)	0.91
HNSC	0.66 (0.46-0.95)	0.027	0.91 (0.86-0.96)	0.00076
LIHC	0.72 (0.41-1.3)	0.26	0.95 (0.84-1.1)	0.34
LUAD	0.69 (0.47-0.99)	0.045	0.9 (0.84-0.97)	0.0073
LUSC	1.1 (0.77-1.5)	0.63	1 (0.96-1.1)	0.52
READ	0.83 (0.34-2.1)	0.69	0.96 (0.83-1.1)	0.54
SKCM	0.43 (0.3-0.62)	4.20E-06	0.93 (0.88-0.98)	0.0061
STAD	0.79 (0.41-1.5)	0.48	1 (0.93-1.2)	0.47

Supplementary Data 1. All potential targets of MIR650

Supplementary Data 2. Molecular markers for each cell type.

1. Mayakonda, A., et al., *Maftools: efficient and comprehensive analysis of somatic variants in cancer.* Genome Res, 2018. **28**(11): p. 1747-1756.
2. Riaz, N., et al., *Tumor and Microenvironment Evolution during Immunotherapy with Nivolumab.* Cell, 2017. **171**(4): p. 934-949.e16.
3. Zhang, J., et al., *The International Cancer Genome Consortium Data Portal.* Nat Biotechnol, 2019. **37**(4): p. 367-369.
4. Cloughesy, T.F., et al., *Neoadjuvant anti-PD-1 immunotherapy promotes a survival benefit with intratumoral and systemic immune responses in recurrent glioblastoma.* Nature medicine, 2019. **25**(3): p. 477-486.
5. Chakravarthy, A., et al., *Pan-cancer deconvolution of tumour composition using DNA methylation.* Nat Commun, 2018. **9**(1): p. 3220.
6. Carter, S.L., et al., *Absolute quantification of somatic DNA alterations in human cancer.* Nat Biotechnol, 2012. **30**(5): p. 413-21.
7. Qian, J., et al., *A pan-cancer blueprint of the heterogeneous tumor microenvironment revealed by single-cell profiling.* Cell Res, 2020. **30**(9): p. 745-762.
